# The Role of Ligand Exchange in Salen Cobalt Complexes in the Alternating Copolymerization of Propylene Oxide and Carbon Dioxide

**DOI:** 10.3390/ijms252010946

**Published:** 2024-10-11

**Authors:** Sergey A. Rzhevskiy, Olga V. Shurupova, Andrey F. Asachenko, Anna V. Plutalova, Elena V. Chernikova, Irina P. Beletskaya

**Affiliations:** 1 Topchiev Institute of Petrochemical Synthesis, Russian Academy of Sciences, Leninskiy Av., 29, 119991 Moscow, Russia; rs89a@yandex.ru (S.A.R.); shurupovao@yandex.ru (O.V.S.); 2 Faculty of Chemistry, Lomonosov Moscow State University, Lenin Hills, 1, Bld. 3, 119991 Moscow, Russia; annaplutalova@gmail.com (A.V.P.); beletska@org.chem.msu.ru (I.P.B.)

**Keywords:** ring-opening copolymerization, polycarbonates, carbon dioxide, salen complexes of cobalt, living polymerization

## Abstract

A comparative study of the copolymerization of racemic propylene oxide (PO) with CO_2_ catalyzed by racemic (salcy)CoX (salcy = *N*,*N*′-bis(3,5-di-*tert*-butylsalicylidene)-1,2-diaminocyclohexane; X = perfluorobenzoate (OBzF_5_) or 2,4-dinitrophenoxy (DNP)) in the presence of a [PPN]Cl ([PPN] = bis(triphenylphosphine)iminium) cocatalyst is performed in bulk at 21 °C and a 2.5 MPa pressure of CO_2_. The increase in the nucleophilicity of an attacking anion results in the increase in the copolymerization rate. Racemic (salcy)CoX provides a high selectivity of the copolymerization, which can be higher than 99%, and the living polymerization mechanism. Poly(propylene carbonate) (PPC) with bimodal molecular weight distribution (MWD) is formed throughout copolymerization. Both modes are living and are characterized by low dispersity, while their contribution to MWD depends on the nature of the attacking anion. The racemic (salcy)CoDNP/[PPN]DNP system is found to be preferable for the production of PPC with a high yield and selectivity.

## 1. Introduction

The modern challenge in polymer chemistry is the development of polymers derived from sustainable resources, which will yield biodegradable polymers with comparable cost-effectiveness and properties to those of petroleum-based polymers [1,2,3,4,5,6]. Among them, polycarbonates synthesized through the copolymerization of epoxides and carbon dioxide have attracted much attention over the last two decades [6,7,8,9,10,11,12,13,14,15,16,17,18,19]. In particular, these come from the usage of carbon dioxide, which is a waste product from different energy sectors with a negative environmental impact. Additionally, its utilization through its incorporation into polymer chains constitutes an important role in the capturing of carbon, positively reducing greenhouse gas emissions.

**Scheme 1:**
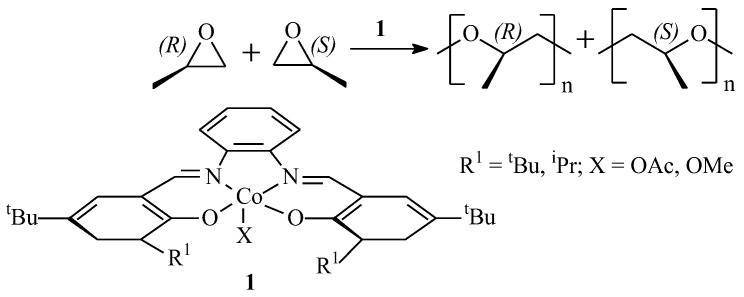
Ring-opening polymerization of (*rac*)-PO in the presence of complex **1** [35,36].

Since the discovery by Inoue in 1969 [20], there have been tremendous advances in the synthesis of polycarbonates, as well as the study of their properties and applications [21,22,23,24,25]. Different catalysts and cocatalysts that can provide regio- and stereoselectivity to the polycarbonates formed, production of polycarbonates at ambient pressures or temperatures, etc. have been developed [26,27]. Salen complexes of Co(III) are rather popular in the copolymerization of CO_2_ and terminal epoxides, e.g., propylene oxide (PO), due to their ease of synthesis and the great variety in the structure of substituents and axial ligands [27,28,29,30,31,32,33,34]. Similar complexes in the combination of ionic cocatalyst salts have previously been found to be highly effective in the homopolymerization of terminal epoxides [35]. For example, Coates et al. [36] reported the first catalyst (complex **1**; X = OAc; R^1^ = ^t^Bu) that was capable of polymerizing (*rac*)-PO to exclusively regioregular (*rac*)-isotactic poly(propylene oxide), obtaining a high yield with a number average molar mass *M_n_* above 5 × 10^4^ kDa (Scheme 1).

**Scheme 2:**
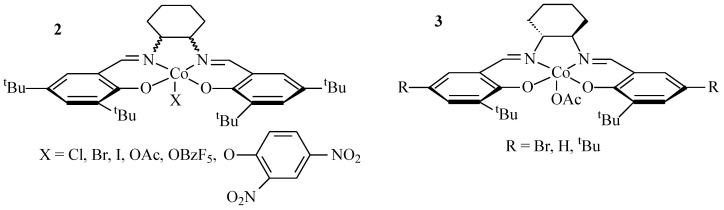
The structure of (salcy)Co^III^X complexes used for the copolymerization of PO and CO_2_ [32,40].

Jacobsen et al. [37,38] have shown that (salcy)Co^III^ carboxylates (salcy = *N*,*N*′-bis(3,5-di-*tert*-butylsalicylidene)-1,2-diaminocyclohexane) can also efficiently ring-open epoxides. At the same time, (salcy)Co^III^X complexes **2**, **3**, and (salen)Co^III^X (complex **1**; R^1^ = ^t^Bu; X = Br) were successfully applied in PO/CO_2_ copolymerization [31,39] (Scheme 2).

These catalysts have provided high selectivity and regioselectivity to polymers. For example, the copolymerization of (*rac*)-PO in the presence of complex **3**, with [PO]/[catalyst] = 500, 5.5 MPa, and 25 °C, resulted in the formation of polypropylene carbonate with *M_n_* = (3–12) × 10^3^; Đ = 1.2–1.6, with selectivity (poly(propylene carbonate)—PPC), and carbonate linkages above 99%. A comprehensive study of ligand modification with salen-type cobalt complexes has revealed that the (salcy) ligand provides better activity and higher regiocontrol [31]. Copolymerization performed under air-free and ambient conditions (traces of water) has no effect on selectivity, while it does affect molecular weight distribution (MWD). The nature of the axial ligand also has an effect on the polymerization rate, and for (*R*,*R*)-(salcy)Co^III^X complex **2**, turn-over frequency (TOF) decreases in the range of X: Br > OBzF_5_ > OAc > Cl > I (where OBzF_5_ is perfluorobenzoate and OAc is acetate). The addition of an ionic cocatalyst can improve the catalytic activities of the complexes. However, its action depends on the nature of the anion and for [PPN]Y ([PPN] = bis(triphenylphosphine)iminium), the activity decreases in the range of Y: Cl > OBzF_5_ > BPh_4_. Simultaneously, the addition of a cocatalyst results in the narrowing of the MWD of the polymer formed. The stereochemistry of both the catalyst and PO has an impact on the catalytic activity of the complex and polymer microstructure. For (*R*,*R*)-(salcy)CoBr, both turn-over frequency (TOF) and regioselectivity decrease in the order of (*S*)-PO > (*rac*)-PO > (*R*)-PO, while the stereochemistry of PO has no effect on the polymerization kinetics and chain microstructure of (*rac*)-(salcy)CoBr. The correlation between the chirality of PO, the catalyst, and the chain microstructure can be seen in Table 1. The (*R*,*R*)-catalyst makes fewer regioerrors when only (*S*)-PO is present. The authors assume that the preferred reaction pathway is the sterically favored attack at the methylene carbon of PO.

**Scheme 3:**
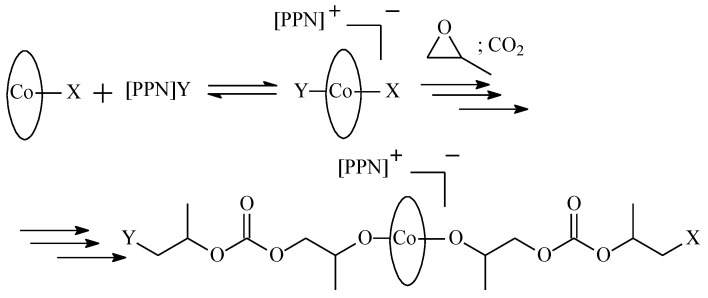
Proposed mechanism for the copolymerization of PO and CO_2_ in the presence of (salcy)CoX and [PPN]Y.

The proposed mechanism involves the coordination of the Lewis acid to the metal center in the axial site, *trans* to the propagating species, resulting in an improved electronic environment for propagation (Scheme 3). The anion of the ionic cocatalyst [PPN]Y is capable of the polymerization initiation resulting in the propagation of the two polymer chains simultaneously from each side of the Co-salcy plane. These propagating species in their turn can dissociate from the metal center during the copolymerization. The similar mechanism was proposed by Inoue et al. for aluminum porphyrins involving ammonium and phosphonium salts [40].

Other features are observed for binary system (*R*,*R*)-salcyCoX bearing a low nucleophilic or/and sterically hindered axial X group (such as 2,4-dinitrophenoxy (DNP)) and using strong base 7-methyl-1,5,7-triazabicyclo[4.4.0]dec-5-ene (MTBD) [41]. Kinetic analysis at 0.6–1.5 MPa and 25 °C has revealed that the CO_2_/(*rac*)-PO copolymerization catalyzed by (*R*,*R*)-(salcy)CoDNP and MTBD (or [PPN]Cl) is characterized by an induction period, whose duration increased with the decrease in both catalyst and cocatalyst concentrations. Additionally, the number of the PPC chains is independent of the initial cobalt complex concentration and is proportional to the concentration of the cocatalyst. In this case, it is assumed that the mechanism involves the coordination of MTBD or [PPN]Cl to the cobalt complex metal center. However, the cocatalyst plays an initiator role in polymer chain growth, while X does not participate in the initiation, which was confirmed by a chain-end group analysis. The activation of the CO_2_ occurred by nucleophilic attack of the –OCH–(CH_3_)CH_2_–MTBD^+^ or –OCH–(CH_3_)CH_2_–[PPN]^+^ species at the Lewis acid carbon atom of the CO_2_ along with the weak interaction between the central metal ion of the cobalt complex and the lone pairs of one oxygen atom of the CO_2_. The dissociation of the propagating carboxylate from the metal center is a much faster process than propagation, and the free propagating carboxylate can serve as a nucleophile attacking a cobalt-coordinated epoxide. However, this conclusion contradicts the results of more recent studies [42], in which a DNP moiety was detected as a terminal group of the polypropylene carbonate, confirming its ability to initiate the polymerization.

The similar observations described by Lu et al. [41] were outlined by Chukanova et al., who have studied the copolymerization of the (*rac*)-PO with CO_2_ in the presence of the (*R*,*R*)-(salcy)CoDNP and the (*R*,*R*)-(salcy)CoCl as a catalyst and [PPN]Cl as a cocatalyst in bulk or in CH_2_Cl_2_ solution at 0.5 MPa and 20 °C [32,33,34]. The duration of the induction period also increased with the decrease in the PO concentration. The reaction order with respect to the PO and catalyst concentration was equal to 1 at [PO]/[catalyst] > 2750 in accordance with the proposed mechanism. However, at lower catalyst concentrations the reaction order with respect to catalyst was close to 2 indicating the change in the mechanism of initiation.

The shape of the polycarbonate MWDs formed in the presence of (salcy)CoX complexes depends on the polymerization conditions. In most cases, the authors have ignored the shape of MWD, specifying *M_n_* and dispersity Đ_M_. However, Sugimoto et al. [43] (cyclohexene oxide—chlorocobalt porphyrin—cocatalyst) and Chukanova et al. [34] (PO—(*R*,*R*)-(salcy)CoDNP—[PPN]Cl) have described the bimodal MWD of the polycarbonate. The transformation of MWD throughout polymerization was described for the (*R,R*)-(salcy)CoDNP—[PPN]Cl system [34]. In this case, the *M_n_* of each mode increased up to 20% of monomer conversion, indicating the presence of living chains of two types. The cause of the bimodal MWD remained unclear. For example, Chukanova et al. [34] explains it by the simultaneous growth of two polymer molecules at the same catalytic center, but it contradicts the supposed mechanism above. In some cases, the bimodal MWD is explained by the presence of traces of water terminating the propagating carboxylate [31]. However, formed macromolecules are unable to participate in the polymerization and are “dead”.

Thus, the aim of the present work is to take a fresh look at the (salcy)CoX catalyst and the [PPN]Y cocatalyst of the PO/CO_2_ copolymerization in the wide range of PO conversions and to compare the influence of ligands in Co catalyst and anions in the [PPN]Y cocatalyst on the copolymerization features and the PPC molecular characteristics. We have demonstrated that the nucleophilicity of anion in the cocatalyst plays an important role in the kinetics of initiation and propagation reactions.

## 2. Results and Discussion

### 2.1. Polymer Synthesis

**Figure 1:**
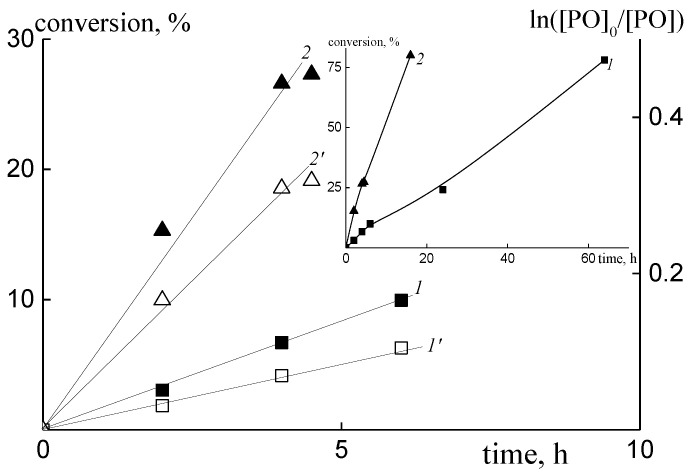
Dependence of the PO conversion (1, 2) and of the ln([PO]_0_/[PO]) (1′, 2′) on the reaction time for the PO/CO_2_ copolymerization in the presence of (*rac*)-(salcy)CoX and [PPN]Cl at 2.5 MPa of CO_2_, 21 °C; for other experimental details, see Table 2. X = OBzF_5_ (1, 1′) and DNP (2, 2′).

Figure 1 presents the dependence of the monomer conversion on the polymerization time for the (*rac*)-PO/CO_2_ copolymerization in the presence of (*rac*)-(salcy)CoOBzF_5_ (1) and (*rac*)-(salcy)CoDNP (2) using the [PPN]Cl as the cocatalyst. The induction period is probably rather short and it cannot be detected for both axial X groups, OBzF_5_ and DNP, under the chosen conditions. The steady state is achieved rather rapidly, and the polymerization kinetics is described by the first-order law with respect to the monomer (lines 1′ and 2′) representing the typical features of living polymerization. However, the deviation of the first-order law kinetics is observed with the further increase in the polymerization duration, indicating a violation of the living polymerization mechanism (inset in Figure 1). It is important to note that the polymerization rate for the copolymerization catalyzed by (*rac*)-(salcy)CoDNP is about 4 times higher than for the copolymerization catalyzed by (*rac*)-(salcy)CoOBzF_5_. Interestingly, for (*R*,*R*)-(salcy)CoDNP and (*R*,*R*)-(salcy)CoOBzF_5_ the kinetics are similar at [PO]/[Cat]/[[PPN]Cl] = 2000/1/1 and 1.5 MPa of the CO_2_ [41]. With the increase in the duration of the polymerization, turn-over number (TON) increases progressively, while TOF is kept about constant up to 10–20% of PO conversion, and then decreases 1.5–2.0 times for the (*rac*)-(salcy)CoOBzF_5_ and 1.3 times for (*rac*)-(salcy)CoDNP (Table 2). Selectivity of PPC over propylene carbonate (PC) is above 95% at conversions up to 10% for (*rac*)-(salcy)CoOBzF_5_ and 25% for (*rac*)-(salcy)CoDNP. However, at higher conversions the accumulation of PC occurs, resulting in the decrease in selectivity.

### 2.2. Copolymer Microstructure

**Scheme 4:**
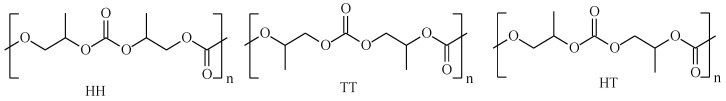
The head-to-head (HH), tail-to-tail (TT), and head-to-tail (HT) junctions for PPC.

According to ^1^H NMR spectroscopy, the carbonate linkages in the polymer prevail throughout polymerization (>99%). The amount of head-to-head (HH), tail-to-tail (TT), and head-to-tail (HT) units (Scheme 4) as well as the ratio of mm:rr:mr triads can be estimated from the ^13^C NMR spectra.

**Figure 2:**
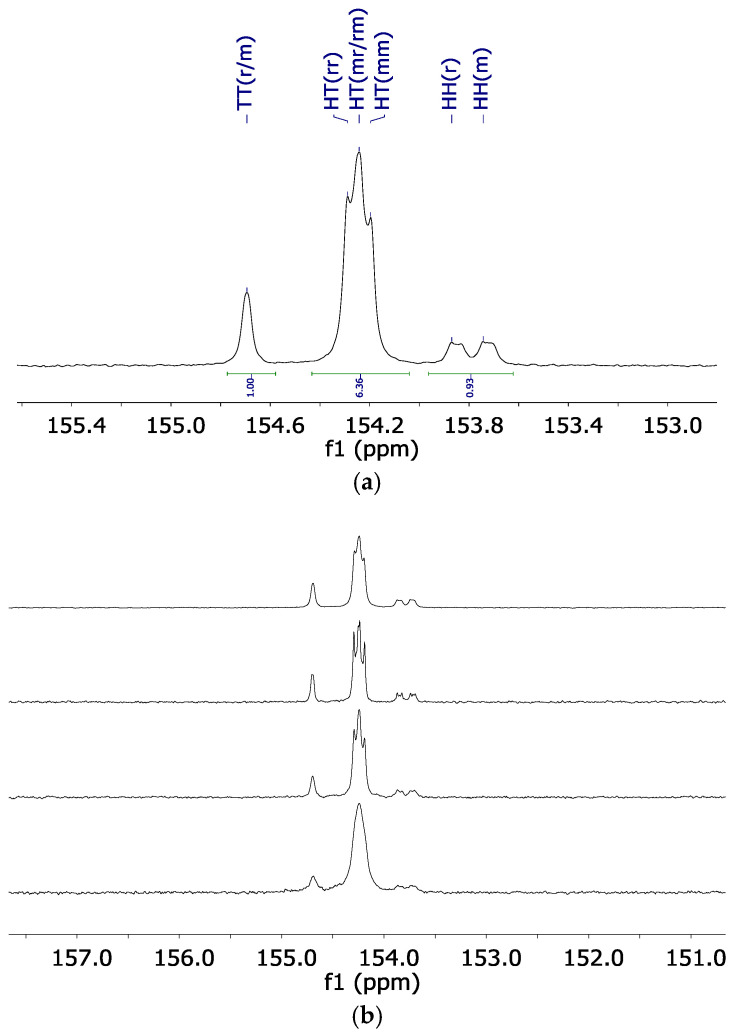
Carbonyl region of the ^13^C NMR spectra of PPC generated from the copolymerization of the (*rac*)-PO/CO_2_ in the presence of (*rac*)-(salcy)CoOBzF_5_ and [PPN]Cl (**a**) at 64 h; (**b**) throughout polymerization, from top to bottom 64, 24, 6, and 2 h.

Figure 2a presents the ^13^C NMR spectrum of PPC in the carbonyl region. The signal at ~154.8 ppm corresponds to TT connectivity, at ~154.2–154.3 ppm to HT connectivity, and at ~153.8–153.9 ppm to HH connectivity. According to previous studies [31], the carbonyl resonances corresponding to the four HT triads ([rr], [mr], [rm], and [mm]) are observed at 154.35, 154.31, 154.29, and 154.25 ppm, while the carbonyl resonances corresponding to the TT [r] and [m] and HH [r] and [m] dyads are observed at 154.76, 154.75, 153.93, and 153.76, respectively. Figure 2b displays the transformation of the carbonyl region of the ^13^C NMR spectra for PPC formed in the presence of (*rac*)-(salcy)CoOBzF_5_ and [PPN]Cl throughout polymerization.

**Figure 3:**
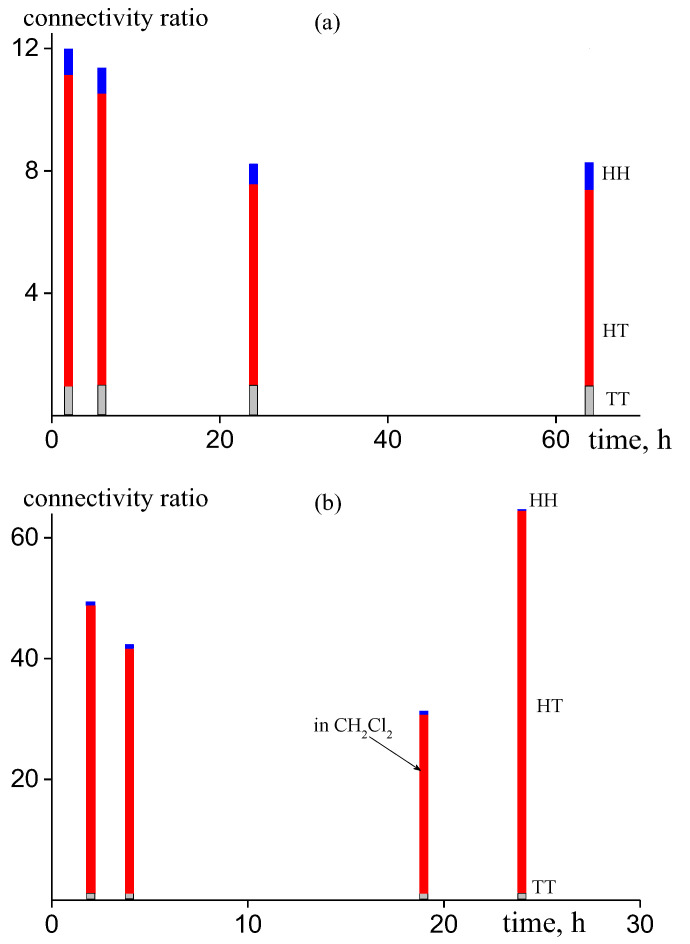
The molar ratio of TT, HT, and HH connectivities in PPC formed in the copolymerization of the (*rac*)-PO/CO_2_ at various polymerization times in the presence of (*rac*)-(salcy)CoOBzF_5_/[PPN]Cl (**a**) and (*rac*)-(salcy)CoDNP/[PPN]Cl (**b**).

Both complexes provide high regioselectivity and the head-to-tail junctions are formed predominantly (Figure 3). However, the proportion of the head-to-tail junctions is 5–8 times higher in the case of complexes with the DNP ligand. The conduction of the copolymerization in CH_2_Cl_2_ results in the decrease in the regiocontrol. For both complexes HT triads show similar distributions; however, in the case of (*rac*)-(salcy)CoOBzF_5_ the molar fraction of [mr]/[rm] triads is slightly higher than that of [rr] and [mm] triads. Thus, PC generated from (*rac*)-PO and (*rac*)-(salcy)CoX is atactic.

### 2.3. Molecular Weight Distribution of PPC

**Figure 4:**
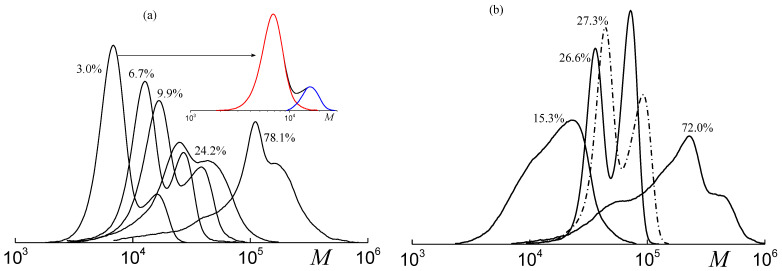
MWDs of the polymers formed at various PO conversions in the PO/CO_2_ copolymerization in the presence of (*rac*)-(salcy)CoOBzF_5_/[PPN]Cl (**a**) and (*rac*)-(salcy)CoDNP/[PPN]Cl (**b**). Conversions are given in the figure. Red and blue lines illustrates the computational simulation of separation of the peaks.

**Figure 5:**
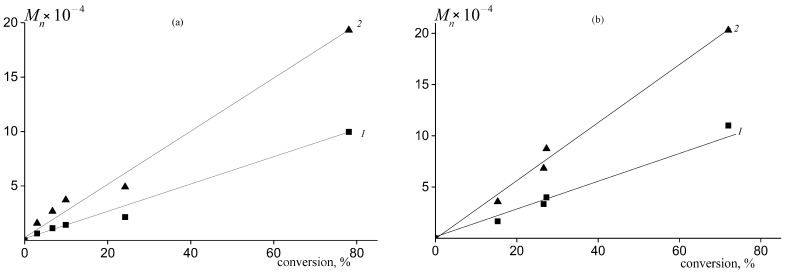
Conversion dependences of *M_n_* corresponding to low-molecular-weight (1) and high-molecular-weight chains (2) produced in the PO/CO_2_ copolymerization in the presence of (*rac*)-(salcy)CoOBzF_5_/[PPN]Cl (**a**) and (*rac*)-(salcy)CoDNP/[PPN]Cl (**b**).

Molecular weight distribution of the PPC is bimodal at the initial and the middle conversions of PO for both complexes used, indicating the presence of two types of propagating chains with the different activities (Figure 4). With the increase in PO conversion, both modes shift to the high-molecular-weight region, confirming the living nature of both types of macromolecules. However, at high conversions MWDs of the polymers become broader for both systems due to the appearance of additional low- and high-molecular-weight modes. It may be supposed that the increase in the viscosity and the decrease in the diffusion of the macromolecules may cause the deviation from the living mechanism. The separation of the peaks (see inset in Figure 4) allows the MWD estimation for each type of propagating chain. As can be seen from Figure 5, both types of chains are living and their *M_n_* increase linearly with the progress in the monomer conversion. Moreover, the dispersity Đ_M_ of both types of chains is very low and lies in the range of 1.03–1.11. The slopes of the *M_n_* conversion dependences for low-molecular-weight and high-molecular-weight peaks differ for both systems and are ~1200/2400 ((*rac*)-(salcy)CoOBzF_5_/[PPN]Cl) and 1600/2900 ((*rac*)-(salcy)CoDNP/[PPN]Cl) for low-molecular-weight/high-molecular-weight chains, respectively. Supposing that *M_n_* dependence on the PO conversion is defined by a typical equation for living polymerization $M_{n}\sim \;conversion\cdot {\left[\mathrm{PO}\right]}_{0}/{\left[\mathrm{catalyst}\right]}_{0}$, it may be assumed that the concentration of the active species responsible for the formation of high-molecular-weight chains is half that of the concentration of the active species responsible for the formation of low-molecular-weight chains. It is curious that the weight ratio of the peaks corresponding to low- and high-molecular-weight chains (*w*_LMW_/*w*_HMW_) varies for both systems with progress in the PO conversion. For X = OBzF_5_ it decreases from ~5 to ~1.2, while for X = DNP it grows from ~0.8 to ~2.6, confirming the different mechanisms of chain initiation.

### 2.4. Thermal Stability of PPC

**Figure 6:**
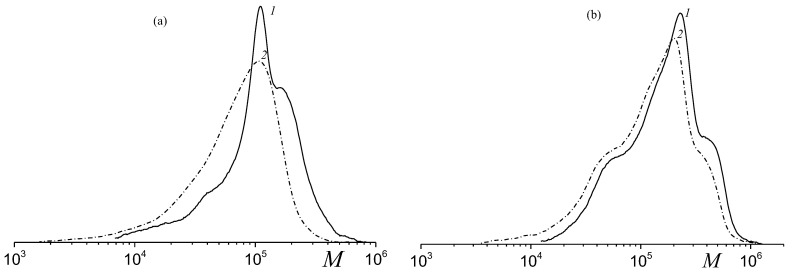
MWDs of the PPC formed in the presence of (*rac*)-(salcy)CoOBzF_5_/[PPN]Cl (**a**) and (*rac*)-(salcy)CoDNP/[PPN]Cl (**b**) before (1) and after (2) heating at 130 °C.

The polymers obtained at the conversion above 70% with two complexes reveal different behaviors on thermal treatment in the inert atmosphere. Figure 6 presents MWDs of the PPC samples before (1) and after (2) heating at 130 °C. PPC obtained in the presence of (*rac*)-(salcy)CoOBzF_5_/[PPN]Cl was subjected to heating for 10 min (Figure 6a), while PPC obtained in the presence of (*rac*)-(salcy)CoDNP/[PPN]Cl was heated for 24 h (Figure 6b). As is seen, PPC synthesized with (*rac*)-(salcy)CoOBzF_5_ degrades more rapidly than PPC synthesized using (*rac*)-(salcy)CoDNP.

**Figure 7:**
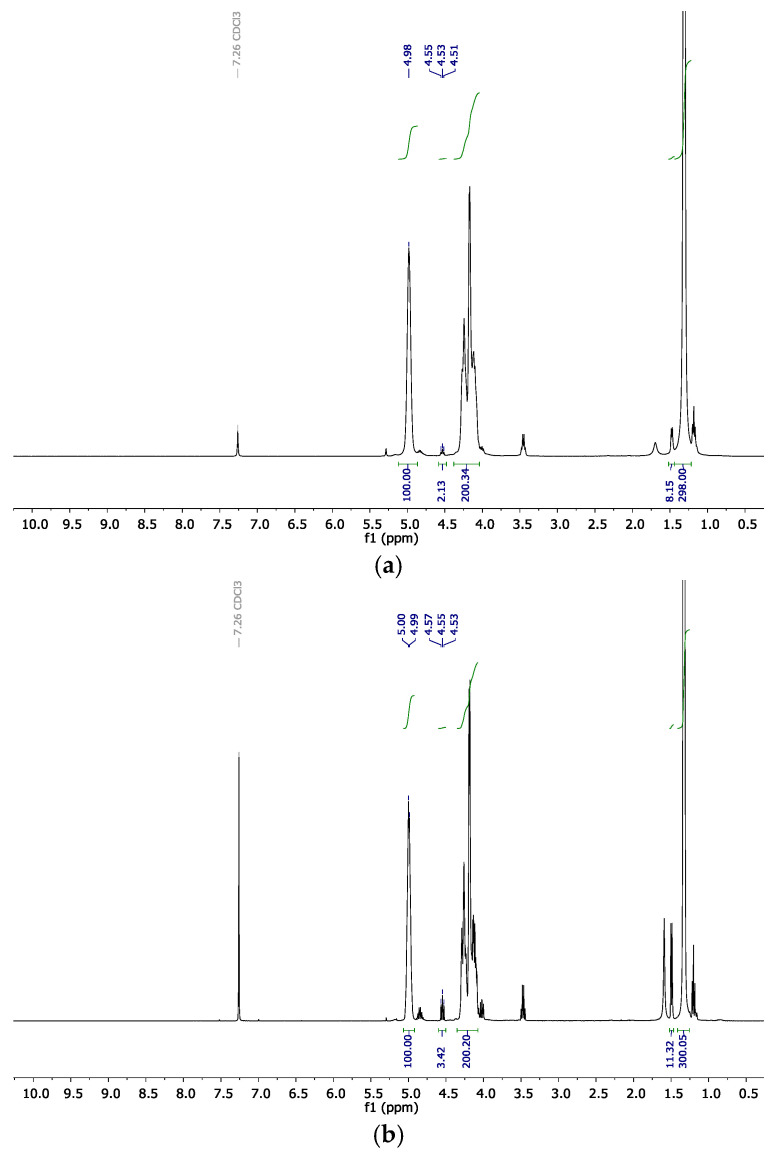

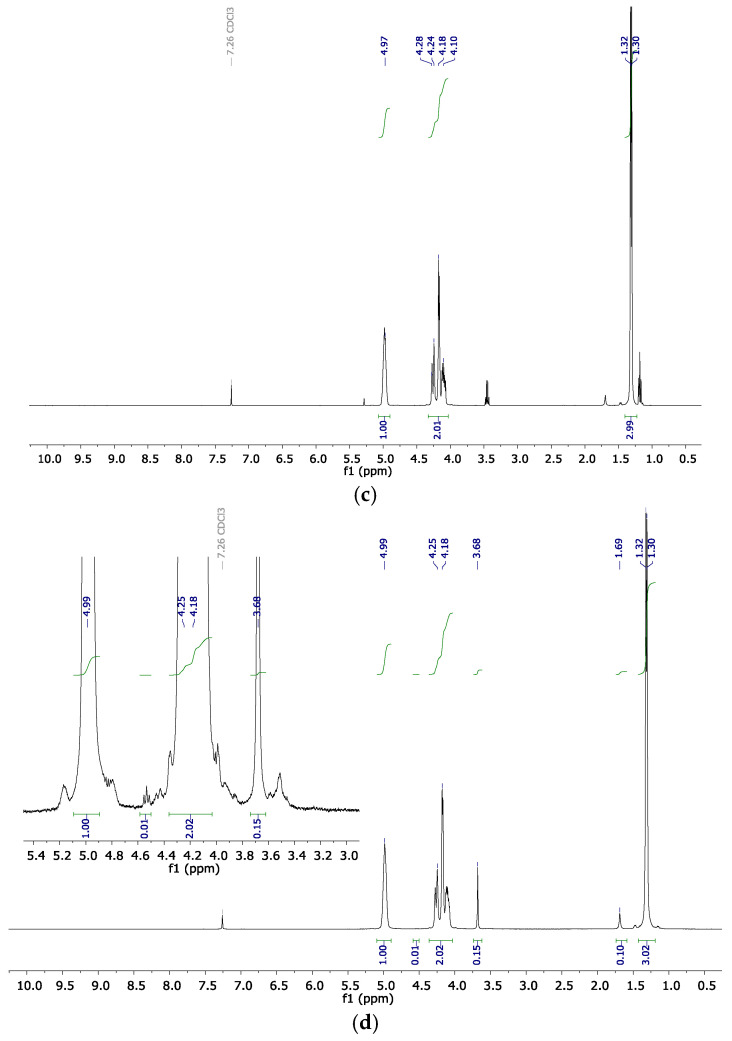
^1^H NMR spectra of PPC formed in the presence of (*rac*)-(salcy)CoOBzF_5_/[PPN]Cl (**a**,**b**) and (*rac*)-(salcy)CoDNP/[PPN]Cl (**c**,**d**) in CDCl_3_ before (**a**,**c**) and after (**b**,**d**) heating at 130 °C.

The polymer degradation is accomplished by backbiting reaction and releasing of the propylene carbonate. Comparing ^1^H NMR spectra of polymers before and after heating (Figure 7) allows concluding that propylene carbonate content in the product of heating is small and is about 3.4 and ~1.0% for PPC formed in the presence of (*rac*)-(salcy)CoOBzF_5_/[PPN]Cl and (*rac*)-(salcy)CoDNP/[PPN]Cl, respectively.

### 2.5. Polymerization Mechanism

**Scheme 5:**
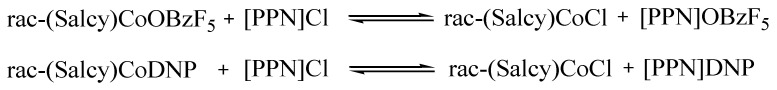
A ligand exchange between (*rac*)-(salcy)CoX (X = OBzF_5_ and DNP) and [PPN]Cl.

Based on these results it may be supposed that the observed differences in kinetics and conversion transformation of MWDs are consequences of the presence of two types of active centers with different reactivities in different ratios in the polymerization system. The reason for the formation of two types of active centers might be a ligand exchange between (*rac*)-(salcy)CoX (X = OBzF_5_ and DNP) and [PPN]Cl leading to the appearance of the two complexes, namely (*rac*)-(salcy)Co X and (*rac*)-(salcy)CoCl, and two cocatalysts, [PPN]Cl and [PPN]Y (Scheme 5). The equilibrium concentrations of the complexes might depend on the extent of binding of the initial ligands to the cobalt.

**Scheme 6:**
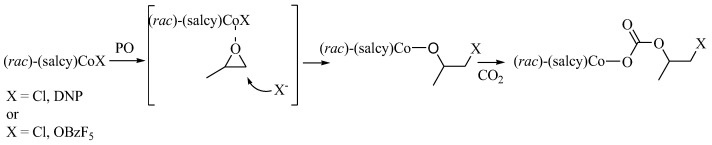
Initiation stage in the PO/CO_2_ copolymerization in the presence of (*rac*)-(salcy)CoX/[PPN]Cl.

During the initiation reaction, the PO molecule is activated by coordination to the metal center of the cobalt complex and the anion of the cocatalyst plays an initiator role resulting in the formation of a metal-bound alkoxide being able to undergo CO_2_ insertion and formation of a metal carbonate (Scheme 6). The higher the nucleophilicity of an attacking anion is, the faster the initiation rate occurs.

**Scheme 7:**
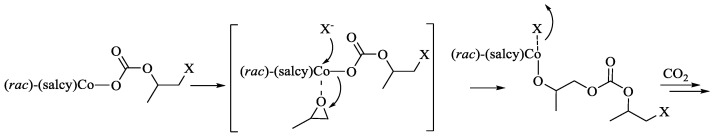
Chain propagation in the PO/CO_2_ copolymerization in the presence of (*rac*)-(salcy)CoX/[PPN]Cl.

The rate of the chain propagation is presumably determined by the intramolecular rearrangement of the Co(III) coordination complex, which contains the growing chain as the counterion and propylene oxide (Scheme 7). We suppose that, similarly to the initiation step, the rate of the intramolecular rearrangement increases with the rise of the nucleophilicity of the anions. We suppose that, among the selected anions, the steric effects are less important than the nucleophilicity. Thus, the presence of two complexes of Co and two types of anions might be responsible for the observed kinetic features of the polymerization.

To confirm this assumption we have prepared additionally three initiating systems, namely (*rac*)-(salcy)CoDNP/[PPN]DNP, (*rac*)-(salcy)CoCl/[PPN]Cl, and (*rac*)-(salcy)CoCl/[PPN]DNP, and conducted the copolymerization of PO and CO_2_ in conditions similar to those described in Table 2. Comparison of the data given in Table 2 and Table 3 allows for conclusion that TON and TOF decrease in the order of the Cat/co-Cat systems: (*rac*)-(salcy)CoDNP/[PPN]DNP ≈ (*rac*)-(salcy)CoCl/[PPN]DNP > (*rac*)-(salcy)CoCl/[PPN]Cl > (*rac*)-(salcy)CoDNP/[PPN]Cl >> (*rac*)-(salcy)CoOBzF_5_/[PPN]Cl. Therefore, we can assume that initiating ability decreases in the following order of the anions: DNP > Cl > OBzF_5_.

**Figure 8:**
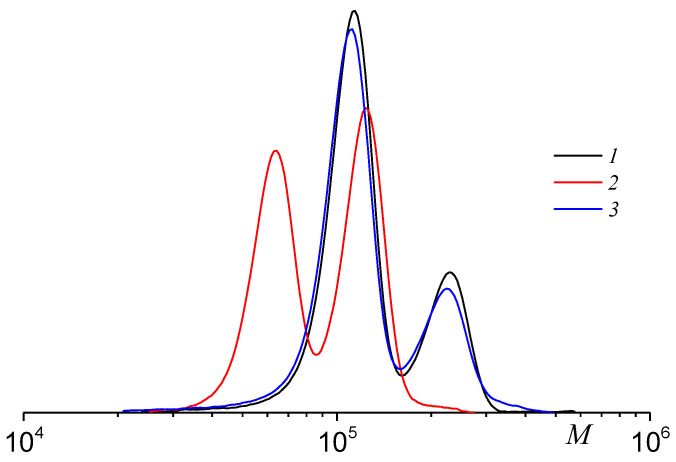
MWDs of the polymers formed after 4 h of copolymerization of PO/CO_2_ in the presence of (*rac*)-(salcy)CoDNP/[PPN]DNP (1), (*rac*)-(salcy)CoCl/[PPN]Cl (2), and (*rac*)-(salcy)CoCl/[PPN]DNP (3).

The MWD of the PPC formed is bimodal, while the dispersities of both modes are narrow, indicating the living mechanism of the PPC formation (Figure 8, Table 3). Thus, the nature of the anion in the structure of both catalyst and [PPN]Y has no impact on the bimodality of MWD. Though the PO conversion is similar, the *M_n_* values corresponding to the low- and high-molecular-weight modes differ by 1.7–1.8 times (Table 3). This difference may result from the various concentrations of the active centers responsible for the formation of the low- and high-molecular-weight modes. The origin of these active centers remains unclear. As was proposed in [31], the simultaneous growth of two macromolecules onto the Co catalyst, which can dissociate from the metal center during the copolymerization, may be responsible for the observed bimodality.

## 3. Materials and Methods

### 3.1. Materials

Methylene chloride (Component-reaktiv, Moscow, Russia, “dry”), and diethyl ether (Component-reaktiv, Moscow, Russia; “reagent grade”) were dried and degassed by passing through a column of activated alumina and by sparging with dry nitrogen. (*rac*)-1,2-Diaminocyclohexane (Sigma-Aldrich, St. Louis, MO, USA, 99%) and 3,5-di-*tert*-butylsalicylaldehyde (Sigma-Aldrich, St. Louis, MO, USA, 99%) were used as received. [PPN]Cl and [PPN]DNP were prepared following a literature procedure [44]. (*Rac*)-propylene oxide (PO) (Sigma-Aldrich, St. Louis, MO, USA, ≥99%) was dried over calcium hydride, distillated under argon atmosphere, and vacuum transferred before use.

### 3.2. Synthesis of the Complexes

All air- or water-sensitive reactions were carried out under dry nitrogen using drybox or standard Schlenk-line techniques. (*rac*)-Salcy ligand was produced from (*rac*)-1,2-diaminocyclohexane and 3,5-di-*tert*-butylsalicylaldehyde according to the known procedure [45].

To synthesize (*rac*)-(salcy)Zn, 1.3 g (5.94 mmol, 1.3 eq) zinc acetate dihydrate was added to a solution of 2.5 g (4.57 mmol) salcy ligand in a mixture of 50 mL methanol and 5 mL triethylamine, stirred overnight, then filtered, washed with methanol, and dried in vacuum. The pale yellow powder was obtained with the yield of 2.69 g (96%).

^1^H NMR (400 MHz, DMSO-d6) δ 8.31 (s, 2H), 7.18 (s, 2H), 7.01 (s, 2H), 3.16 (s, 2H), 1.68–1.09 (m, 44H); ^13^C{H}NMR (101 MHz, DMSO) δ 168.5, 164.9, 140.1, 132.1, 129.0, 126.4, 117.9, 64.5, 35.1, 33.4, 31.5, 29.6, 27.7, 24.0. ESI-HRMS (*m*/*z*): calc. for (C_36_H_52_N_2_O_2_Zn) [M+H]^+^: 609.3393; found: 609.3390.

(*rac*)*-*(Salcy)Co(II) was synthesized through ligand exchange with (*rac*)*-*(salcy)Zn: 1.02 g (4.1 mmol) cobalt(II) acetate tetrahydrate was added to a solution of 2.5 g (4.1 mmol) (*rac*)-(salcy)Zn in 20 mL of THF, stirred overnight, then the precipitate was filtered, washed with methanol, and dried in vacuum. The yield of the product formed as a red powder is 2.2 g (90%).

**Scheme 8:**
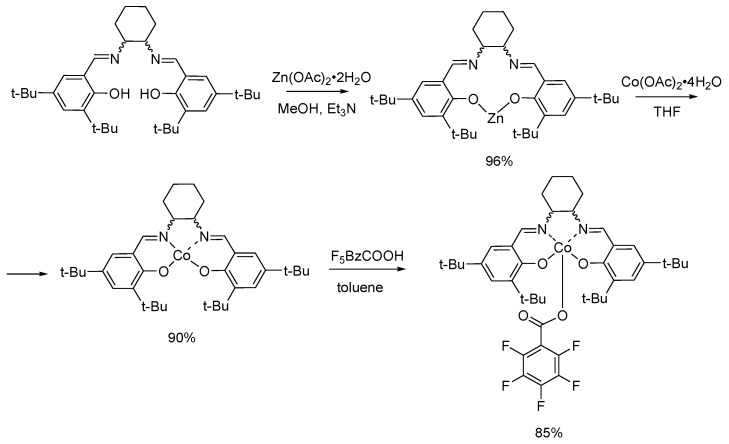
The general scheme of the synthesis of *rac*-(salcy)Co(III)OBzF_5_.

*rac*-(Salcy)Co(III)OBzF_5_ was synthesized according to a modified procedure [32]. A solution of 100 mg (0.165 mol) of (*rac*)-(salcy)Co(II) and 35 mg (0.165 mol) of pentafluorobenzoic acid in 10 mL of CH_2_Cl_2_ was mixed overnight, planted with hexane, and recrystallized from a mixture of CH_2_Cl_2_/petroleum ether. The yield of the product formed as a green powder is 114 mg (85%). The general scheme of the synthesis of *rac*-(salcy)Co(III)OBzF_5_ is given in Scheme 8.

^1^H NMR (400 MHz, DMSO-d6) δ 7.8 (s, 2H), 7.46-7.43 (m, 4H), 3.60 (m, 2H), 3.07–3.05 (m, 2H), 1.99–1.98 (m, 4H), 1.73 (s, 18H), 1.57–1.55 (m, 2H) 1.29 (s, 18H); ^13^C{H} NMR (101 MHz, DMSO) δ 164.5, 162.0, 141.7, 135.8, 129.1, 128.7, 118.5, 69.2, 35.7, 33.4, 31.4, 30.9, 30.3, 29.4, 24.2; ^19^F NMR (376 MHz, DMSO-d6) δ −162.97, −161.23, −143.64.

**Scheme 9:**
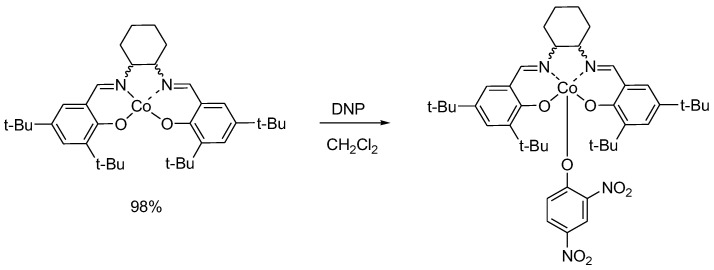
The general scheme of the synthesis of *rac*-(salcy)Co(III)DNP.

*rac*-(Salcy)Co(III)DNP was synthesized according to the modified procedure (Scheme 9) [41,42]. A solution of 1 g (1.65 mmol) of (*rac*)-(salcy)Co(II) and 336 mg (1.826 mmol) of 2,4-dinitrophenol in 100 mL of CH_2_Cl_2_ was mixed for 4 h, the solvent was evaporated, and the resulting precipitate was recrystallized from a mixture of CH_2_Cl_2_/hexane = 1:2 *v*/*v*. The yield of the product formed as a black powder is 900 mg (69%).

^1^H NMR (400 MHz, DMSO-d6) δ 8.59 (d, J = 3.1 Hz, ^1^H), 7.81 (s, 2H), 7.79–7.74 (m, ^1^H), 7.46–7.45 (m, 4H), 6.30 (d, J = 9.8 Hz, ^1^H), 3.61 (d, J = 8.1 Hz, 2H), 3.07 (d, J = 11.1 Hz, 2H), 2.02–1.906 (m, 4H), 1.74 (s, 18H), 1.61–1.57 (m, 2H), 1.30 (s, 18H); ^13^C NMR (101 MHz, DMSO) δ 164.6, 162.0, 141.8, 135.9, 129.2, 128.7, 127.4, 127.2, 125.1, 118.6, 69.2, 35.8, 33.5, 31.5, 30.4, 29.5, 24.3.

### 3.3. Polymerization Procedure

The typical procedure of the polymer synthesis is as follows. The mixture of (*rac*)-(salcy)CoX (X = OBzF_5_, DNP) complex (0.014 mmol) and [PPN]Cl (0.014 mmol) was dissolved in (*rac*)-PO (6 mL, 85.7 mmol, 6000 equiv.) in a 10 mL vial equipped with a Teflon-coated magnetic stir bar. The mixture was allowed to stir until red-brown homogeneous solution was formed. Then, the vial was placed into a pre-dried 100 mL autoclave, which was pressurized to the appropriate pressure with CO_2_. After the required reaction time, the mixture was dissolved in 50 mL of CH_2_Cl_2_ and concentrated in vacuum. The polymer was isolated by precipitation from CH_2_Cl_2_/MeOH (10/1, *v*/*v*) solution in diethyl ether, filtration, and long-term drying in vacuum.

^1^H NMR (400 MHz, Chloroform-d) δ 4.98 (br. s, ^1^H), 4.27–4.10 (m, 2H), 1.31 (d, J = 6.4 Hz, 3H).

^13^C{H} NMR (101 MHz, Chloroform-d) δ 154.7, 154.2, 154.2, 153.8, 153.7, 72.4, 72.1, 69.3, 69.2, 69.0, 16.2.

### 3.4. Instrumentation

NMR spectra were obtained on a Bruker Avance III HD (400 MHz ^1^H, 101 MHz ^13^C, 376 MHz ^19^F; Bruker corp., Billerica, MA, USA). The chemical shifts are frequency referenced relative to the residual undeuterated solvent peaks. Coupling constants J are given in Hertz as positive values regardless of their real individual signs. The multiplicity of the signals is indicated as “s”, “d”, “t”, or “m” for singlet, doublet, triplet, or multiplet, respectively. The abbreviation “br” is given for broadened signals.

High-resolution mass spectrometry was carried out using an AB Sciex TripleTOF 5600+ (SCIEX, Redwood City, CA, USA) equipped with DuoSray ion sources.

The SEC measurements were performed in THF at 40 °C with a flow rate of 1.0 mL/min using a 1260 Infinity II GPC/SEC Multidetector System chromatograph (Agilent, Santa Clara, CA, USA) equipped with two PLgel 5 μm MIXED B columns. The SEC system was calibrated using narrow dispersed linear poly(methyl methacrylate) standards with MW ranging from 0.8 to 2000 kDa.

## 4. Conclusions

Summarizing, in the present research we have performed a comparative study of (*rac*)-PO copolymerization with CO_2_ catalyzed by (*rac*)-(Salcy)CoX (X = OBzF_5_ and DNP) with the cocatalyst [PPN]Y (Y = Cl, DNP) in bulk at room temperature (21 °C) and 2.5 MPa pressure of CO_2_. We have found that the copolymerization rate (PO conversion, TON, TOF), selectivity, molar part of HT junctions, MWs, and thermostability are higher for the PPC synthesized under the (*rac*)-(Salcy)CoDNP catalysis. We assume that the differences in the kinetics behavior of the systems result from the different nucleophilicity of an attacking anion. Additionally, we have supposed that the ligand exchange takes place between the anions of the catalyst and the cocatalyst. This assumption has been proved by experiments with (*rac*)-(Salcy)CoDNP/[PPN]DNP and (*rac*)-(Salcy)CoCl/[PPN]Cl, which had shown that the copolymerization rate decreases in the range of the catalytic systems: (*rac*)-(salcy)CoDNP/[PPN]DNP ≈ (*rac*)-(salcy)CoCl/[PPN]DNP > (*rac*)-(salcy)CoCl/[PPN]Cl > (*rac*)-(salcy)CoDNP/[PPN]Cl >> (*rac*)-(salcy)CoOBzF_5_/[PPN]Cl. For all the systems we observe the living mechanism, which is confirmed by the increase in the *M_n_* with the monomer conversion and the formation of PPC with the narrow MWD. At high PO conversions (~70%) the increase in the viscosity results in the violation of the living mechanism probably due to the low diffusion of active species. All the polymers formed at initial and middle conversions have a bimodal MWD, which probably results from the presence of two active centers with various activities and concentrations. The ratio of these active centers depends on the nature of anions in the catalyst/cocatalyst systems.

Thus, we have demonstrated that all anions, namely DNP, Cl, and OBzF_5_, are capable of initiating the polymerization. However, the (*rac*)-(salcy)CoDNP/[PPN]DNP system is preferable for production of the PPC with high yield and selectivity.

## Figures and Tables

**Table 1:** The influence of the chirality of the catalyst and PO on the polymer microstructure [32].

Catalyst	PO	Microstructure
(*R*,*R*)-(salcy)CoBr	(*rac*)-PO	Regioregular atactic
	(*S*)-PO	Regioregular isotactic
	(*R*)-PO	Regiorandom heterotactic
(*rac*)-(salcy)CoBr	(*R*)-PO	Regioregular isotactic
	(*rac*)-PO	Regioregular syndio-enriched
(*R*,*R*)-(salcy)CoOBzF_5_	(*rac*)-PO	Regioregular iso-enriched
	(*S*)-PO	Regioregular isotactic
(*rac*)-(salcy)CoOBzF_5_	(*R*)-PO	Regioregular isotactic
	(*rac*)-PO	Regioregular atactic

**Table 2:** (*rac*)-(Salcy)CoX-catalyzed copolymerization of the (*rac*)-PO and CO_2_ in the presence of [PPN]Cl.

Catalyst	[PO]/[Cat]	[Cat]/[[PPN]Cl]	Time, h	Selectivity, PPC%	TON	TOF, h^−1^
(*rac*)-(salcy)CoOBzF_5_	6420	0.87	2	>97	195	98
	6630	0.86	4	>97	440	110
	6350	0.86	6	>97	630	105
	6500	0.86	24	>50	1570	65
	6460	0.86	72	>50	5050	79
(*rac*)-(salcy)CoDNP	6170	0.90	2	96.0	950	470
	6480	0.88	4	97.2	1730	430
	7080	0.74	4.5	98.4	1930	430
	6500	0.81	24	>75	4784	199

**Table 3:** (*rac*)-(Salcy)CoX (X = DNP, Cl, OBzF_5_)-catalyzed copolymerization of (*rac*)-PO and CO_2_ in the presence of [PPN]Y (Y = DNP, Cl); duration 4 h, 21 °C, 2.5 MPa.

Cat/Co-Cat	[PO]/[Cat]	Conversion (PO), %	Selectivity, PPC%	TON	TOF, h^−1^	M_n1_/M_n2_, ^(a)^ kDa	Đ_M1_/Đ_M2_ ^(a)^
(*rac*)-(salcy)CoDNP [PPN]DNP	6670	38.2	99.0	2548	637	103.2/220.6	1.05/1.03
(*rac*)-(salcy)CoCl [PPN]Cl	5990	40.4	96.4	2422	605	60.0/121.1	1.04/1.03
(*rac*)-(salcy)CoCl [PPN]DNP	6000	43.0	97.5	2582	645	98.2/220.8	1.07/1.04

^(a)^ M_n1_/M_n2_ and Đ_M1_/Đ_M2_ are the number average molar masses and dispersities of low- and high-molecular-weight modes, respectively.

## Data Availability

The raw data supporting the conclusions of this article will be made available by the authors on request.

## References

[1] Cao H., Wang X. (2021). Carbon dioxide copolymers: Emerging sustainable materials for versatile applications. SusMat.

[2] Artz J., Müller T.E., Thenert K., Kleinekorte J., Meys R., Sternberg A., Bardow A., Leitner W. (2018). Sustainable Conversion of Carbon Dioxide: An Integrated Review of Catalysis and Life Cycle Assessment. Chem. Rev..

[3] Zhu Y., Romain C., Williams C.K. (2016). Sustainable polymers from renewable resources. Nature.

[4] Chen G.Q., Patel M.K. (2012). Plastics derived from biological sources: Present and future: A technical and environmental review. Chem. Rev..

[5] Muthuraj R., Mekonnen T. (2018). Recent progress in carbon dioxide (CO_2_) as feedstock for sustainable materials development: Co-polymers and polymer blends. Polymer.

[6] Grignard B., Gennen S., Jérôme C., Kleij A.W., Detrembleur C. (2019). Advances in the use of CO_2_ as a renewable feedstock for the synthesis of polymers. Chem. Soc. Rev..

[7] Liu Y., Zhou H., Guo J.Z., Ren W.M., Lu X.B. (2017). Completely recyclable monomers and polycarbonate: Approach to sustainable polymers. Angew. Chem. Int. Ed..

[8] Qin Y., Wang X.H. (2010). Carbon dioxide-based copolymers: Environmental benefits of PPC, an industrially viable catalyst. Biotechnol. J..

[9] Darensbourg D.J., Wilson S.J. (2012). What’s new with CO_2_? Recent advances in its copolymerization with oxiranes. Green Chem..

[10] Lu X.B., Darensbourg D.J. (2012). Cobalt catalysts for the coupling of CO_2_ and epoxides to provide polycarbonates and cyclic carbonates. Chem. Soc. Rev..

[11] Paul S., Zhu Y., Romain S., Brooks R., Saini P.K., Williams C.K. (2015). Ring-opening copolymerization (ROCOP): Synthesis and properties of polyesters and polycarbonates. Chem. Commun..

[12] Huang J., Worch J.C., Dove A.P., Coulembier O. (2020). Update and challenges in carbon dioxide-based polycarbonate synthesis. ChemSusChem.

[13] Lu X.B., Ren B.H. (2022). Partners in Epoxide Copolymerization Catalysis: Approach to High Activity and Selectivity. Chin. J. Polym. Sci..

[14] Yang G.W., Wang Y., Qi H., Zhang Y.Y., Zhu X.F., Lu C., Yang L., Wu G.P. (2022). Highly Selective Preparation and Depolymerization of Chemically Recyclable Poly (cyclopentene carbonate) Enabled by Organoboron Catalysts. Angew. Chem. Int. Ed..

[15] Chen C., Gnanou Y., Feng X. (2023). Ultra-Productive Upcycling CO_2_ into Polycarbonate Polyols via Borinane-Based Bifunctional Organocatalysts. Macromolecules.

[16] Zhao M., Zhu S., Zhang G., Wang Y., Liao Y., Xu J., Zhou X., Xie X. (2023). One-Step Synthesis of Linear and Hyperbranched CO_2_-Based Block Copolymers via Organocatalytic Switchable Polymerization. Macromolecules.

[17] Naumann S. (2023). Borane catalysis for epoxide (co)polymerization. Polym. Chem..

[18] Wang Y., Liu Z., Guo W., Zhang C., Zhang X. (2023). Phosphine-Borane Frustrated Lewis Pairs for Metal-Free CO_2_/Epoxide Copolymerization. Macromolecules.

[19] Zhou X., Zhai Y., Ren K., Cheng Z., Shen X., Zhang T., Bai Y., Jia Y., Hong J. (2023). Life cycle assessment of polycarbonate production: Proposed optimization toward sustainability. Resour. Conserv. Recycl..

[20] Inoue S., Koinuma H., Tsuruta T. (1969). Copolymerization of carbon dioxide and epoxide with organometallic compounds. Macromol. Chem..

[21] Trott G., Saini P.K., Williams C.K. (2016). Catalysts for CO_2_/epoxide ring-opening copolymerization. Philos. Trans. R. Soc. A.

[22] Kernbichl S., Rieger B., Reina T.R., Arellano-Garcia H., Odriozola J.A. (2021). Aliphatic Polycarbonates Derived from Epoxides and CO_2_. Engineering Solutions for CO_2_ Conversion.

[23] Liang X., Tan F., Zhu Y. (2021). Recent Developments in Ring-Opening Copolymerization of Epoxides with CO_2_ and Cyclic Anhydrides for Biomedical Applications. Front. Chem..

[24] Song B., Qin A., Tang B.Z. (2022). Syntheses, properties, and applications of CO_2_-based functional polymers. Cell Rep. Phys. Sci..

[25] Lidston C.A.L., Severson S.M., Abel B.A., Coates G.W. (2022). Multifunctional Catalysts for Ring-Opening Copolymerizations. ACS Catal..

[26] Nakano K., Hashimoto S., Nakamura M., Kamada T., Nozaki K. (2011). Stereocomplex of Poly (propylene carbonate): Synthesis of Stereogradient Poly(propylene carbonate) by Regio- and Enantioselective Copolymerization of Propylene Oxide with Carbon Dioxide. Angew. Chem. Int. Ed..

[27] Lu X.B., Wang Y. (2004). Highly Active, Binary Catalyst Systems for the Alternating Copolymerization of CO_2_ and Epoxides under Mild Conditions. Angew. Chem. Int. Ed..

[28] Paddock R.L., Nguyen S.T. (2004). Chiral (salen) Co^III^ catalyst for the synthesis of cyclic carbonates. Chem. Commun..

[29] Cohen C.T., Thomas C.M., Peretti K.L., Lobkovsky E.B., Coates G.W. (2006). Copolymerization of cyclohexene oxide and carbon dioxide using (salen) Co(III) complexes: Synthesis and characterization of syndiotactic poly (cyclohexene carbonate). Dalton Trans..

[30] Cohen C.T., Coates G.W. (2006). Alternating copolymerization of propylene oxide and carbon dioxide with highly efficient and selective (salen) Co(III) catalysts: Effect of ligand and cocatalyst variation. J. Polym. Sci. Polym. Chem..

[31] Cohen C.T., Chu T., Coates G.W. (2005). Cobalt Catalysts for the Alternating Copolymerization of Propylene Oxide and Carbon Dioxide:  Combining High Activity and Selectivity. J. Am. Chem. Soc..

[32] Chukanova O.M., Belov G.P. (2017). Reaction between carbon dioxide and propylene oxide catalyzed by cobalt and chromium porphyrin complexes: The effect of reaction conditions on the reaction rate. Kinet. Catal..

[33] Chukanova O.M., Belov G.P. (2016). Effect of the ligand nature in cobalt complexes on the selectivity of the reaction of carbon dioxide and propylene oxide. Kinet. Catal..

[34] Chukanova O.M., Perepelitsina E.O., Belov G.P. (2014). The influence of reagent concentration on the kinetics of carbon dioxide-propylene oxide copolymerization in the presence of a cobalt complex. Polym. Sci. Ser. B.

[35] Ajiro H., Peretti K., Lobkovsky E.B., Coates G.W. (2009). On the mechanism of isospecific epoxide polymerization by salen cobalt(III) complexes: Evidence for solid-state catalysis. Dalton Trans..

[36] Peretti K.L., Ajiro H., Cohen C.T., Lobkovsky E.B., Coates G.W. (2005). A Highly Active, Isospecific Cobalt Catalyst for Propylene Oxide Polymerization. J. Am. Chem. Soc..

[37] Tokunaga M., Larrow J.F., Kakiuchi F., Jacobsen E.N. (1997). Asymmetric Catalysis with Water: Efficient Kinetic Resolution of Terminal Epoxides by Means of Catalytic Hydrolysis. Science.

[38] Nielsen L.P.C., Stevenson C.P., Blackmond D.G., Jacobsen E.N. (2004). Mechanistic Investigation Leads to a Synthetic Improvement in the Hydrolytic Kinetic Resolution of Terminal Epoxides. J. Am. Chem. Soc..

[39] Qin Z., Thomas C.M., Lee S., Coates G.W. (2003). Cobalt-Based Complexes for the Copolymerization of Propylene Oxide and CO_2_: Active and Selective Catalysts for Polycarbonate Synthesis. Angew. Chem. Int. Ed..

[40] Aida T., Inoue S. (1996). Metalloporphyrins as Initiators for Living and Immortal Polymerizations. Acc. Chem. Res..

[41] Lu X.-B., Shi L., Wang Y.-M., Zhang R., Zhang Y.-J., Peng X.-J., Zhang Z.-C., Li B. (2006). Design of Highly Active Binary Catalyst Systems for CO_2_/Epoxide Copolymerization:  Polymer Selectivity, Enantioselec-tivity, and Stereochemistry Control. J. Am. Chem. Soc..

[42] Zhuang X., Oyaizu K., Niu Y., Koshika K., Chen X., Nishide H. (2010). Synthesis and electrochemistry of Schiff base cobalt(III) complexes and their catalytic activity for copolymerization of epoxide and carbon dioxide. Macromol. Chem. Phys..

[43] Sugimoto H., Kuroda K. (2008). The Cobalt Porphyrin−Lewis Base System:  A Highly Selective Catalyst for Alternating Copolymerization of CO_2_ and Epoxide under Mild Conditions. Macromolecules.

[44] Kukushkin V.Y., Moiseev A.I. (1990). Convenient synthesis of μ-nitridobis(triphenylphosphonium) chloride ([PPN]Cl) with contribution of PCl_5_ as chlorinating (PCl_5_ + PPh_3_) or deoxygenating (PCl_5_ + OPPh_3_) reagent. Inorg. Chim. Acta.

[45] Larrow J.F., Jacobsen E.N. (1998). (R,R)-N,N′-Bis(3,5-di-tert-Butylsalicylidene)-1,2-Cyclohexanediamino Manganese(III) Chloride, A Highly Enantioselective Epoxidation Catalyst. Org. Synth. Coll..

